# Cognitive Aspects of the Motor Planning Stage of Gait: Effects of Aging and Parkinson’s Disease

**DOI:** 10.1093/geroni/igaf122.2557

**Published:** 2025-12-31

**Authors:** Jeffrey Hausdorff, Galia Shaham, Irina Galperin, Amit Salomon, Eran Gazit, Aron Buchman, James Richardson

**Affiliations:** Tel Aviv Sourasky Medical Center, Tel Aviv, Tel Aviv, Israel; Tel Aviv University, Tel Aviv, Tel Aviv, Israel; Tel Aviv Sourasky Medical Center, Tel Aviv, Tel Aviv, Israel; Tel Aviv Sourasky Medical Center, Tel Aviv, Tel Aviv, Israel; Tel Aviv Sourasky Medical Cetner, Tel Aviv, Tel Aviv, Israel; Rush University, Chicago, Illinois, United States; University of Michigan, Ann Arbor, Michigan, United States

## Abstract

The response to a request to walk involves a motor planning phase followed by an execution phase. The initial phase of gait initiation, specifically the time to anticipatory postural adjustment (APA), can be viewed as a form of reaction time. However, it is not clear how to characterize the cognitive processes involved in this stage. To address this question, time-to-APA, simple and complex upper limb visuomotor reaction time (SRT, CRT), cognitive, and motor performance were evaluated in 27 people with Parkinson’s disease (PD), 31 older adults (OA), and 34 young adults (YA). Our results showed that time-to-APA was significantly longer than SRT in all three groups (p < 0.001), indicating a more complex cognitive process. In YA, time-to-APA was significantly shorter than CRT (p < 0.001). In the OA and PD, time-to-APA was not significantly different from CRT. Mixed-effects analysis showed significant time (p < 0.001), group (p = 0.037), and group × time interaction effects (p = 0.002). Among all subjects, time-to-APA, but not APA duration, was associated with the Color-Trails Test (part B: rs = 0.406, p < 0.001). In PD, APA duration was correlated with MDS-UPDRS-part 3 (motor) scores (rs = 0.535, p = 0.004), but time-to-APA was not (p = 0.892). These findings suggest that time-to-APA is a cognitive process that is more complex than a SRT task and shares properties of a CRT task, especially among older adults and people with PD. In PD, this initial movement planning stage is not related to motor impairment, in contrast to APA duration. Further research is necessary to identify the factors underlying this initial stage of gait initiation.

